# Time Heals all Wounds- but Scars Remain. Can Personalized Medicine Help?

**DOI:** 10.3389/fgene.2018.00211

**Published:** 2018-06-22

**Authors:** Saeid Amini-Nik

**Affiliations:** ^1^Sunnybrook Research Institute, Toronto, ON, Canada; ^2^Laboratory Medicine and Pathobiology, University of Toronto, Toronto, ON, Canada; ^3^Division of Plastic Surgery, Department of Surgery, University of Toronto, Toronto, ON, Canada

**Keywords:** personalized medicine, precision medicine, skin scar, keloids, scar, genomics, proteomics, mesenchymal stem cell

New advances in Omics approaches have facilitated the stratification of patients and may identify the patients that would scar more aggressively. These advances may prevent undertreating patients and avoid their exposure to unnecessary treatment with side effects.

Scars have been documented for 3,700 years, yet they are still not entirely preventable. Can personalized medicine help?

In the ancient Egyptian medical text, Edwin Smith Papyrus, along with 47 other cases, scarring was first described (Berman and Bieley, 1995). Despite advancement in the management of several diseases during the last centuries, scarring remains a tremendous clinical challenge to patients and clinicians. The precise incidence and prevalence of scars like keloids are unknown. Keloid incidences have been reported as high as 16% in individuals of Hispanic and African ancestry (Niessen et al., 1999). They are aesthetically disfiguring, functionally debilitating, emotionally distressing, and psychologically damaging, resulting in a significant burden for patients. While our knowledge about scarring continues to advance, we are still puzzled by this fibrodysplastic response.

The increased incidence of scar formation in specific ethnic groups such as blacks, Hispanics points to there being a genetic predisposition to keloid formation (Niessen et al., 1999). In Asians, it is reported that the annual keloid incidence rate is about 0.15% of the general population, with women outnumbering men by a ratio of 1.33 (Sun et al., 2014). Patients with darker skin are 15 times more likely than patients with lighter skin to develop keloids (Miller and Nanchahal, 2005). These all suggest that genetic factors are important in understanding the pathobiology of keloids. However, no responsible genes have yet been identified for Keloids. A genome-wide association study on 517 cases and 2,385 controls, has shown that four SNP loci (rs873549 at 1q41, rs940187, and rs1511412 at 3q22.3, rs8032158 at 15p21.3) associate significantly with keloid development in the Japanese population (Nakashima et al., 2010) and these regions include genes such as *FOXL2, NEDD4*. However, the relevance of these genes in keloid formation is not fully unraveled. Besides the genomic variabilities, epigenetic and transcriptional variability contribute to the diversity of cellular phenotypes in diseases, including scar formation.

An initial insult is necessary to develop scars. This raises the possibility that initial events post injury (e.g., inflammation) are the essential components for the development of scars (e.g., keloid) in an already susceptible individual. If the inflammation fails to subside, and instead, becomes increasingly prominent, it keeps the process of wound healing active which eventually yields pathological scars, which become apparent a few months after the initial insult. Besides inflammatory cells, an essential role for mesenchymal progenitor cells has been extrapolated during normal as well as fibrotic responses (Bielefeld et al., 2013). In the case of keloids, it has also been shown that keloids are populated by mesenchymal progenitor cells (Iqbal et al., 2012). However, it is not clear what cues attract mesenchymal progenitor cell into the healing wounds which generate scars. Nonetheless, wound healing is a complex phenomenon, and it is difficult to attribute scar formation to a specific phase or a specific cell type.

If not excessive scarring, the minimal consequence of a deep skin injury is a “physiologic scar.” Most people recovering from surgery, burn or other traumatic injury are left with some scarring (Amini-Nik et al., 2017). Besides the aesthetic outcomes which are essential for patients, the functional result of skin healing and prevention of disfigurement are necessary. Although novel medications show promising effects *in vitro* and *in vivo* in regard to preventing scar formation, it has yet to be examined in patients (Poon et al., 2012). The differences in responses, using currently approved medications, may be explained by Omics variation, particularly Genomics approaches (Zurada et al., 2006; Kerwin et al., 2014; Sidgwick et al., 2015). Thus, a substantial unmet clinical need for prediction of scar formation and their diverse response to the treatment exists (Amini-Nik et al., 2017). New predictive biomarkers may help clinicians to predict the risk of skin scar formation and then tailor the antifibrotic treatment strategies to each patient. To reach this goal, we need operative scar biobanking of patient tissue, genomics, and proteomics approach to verify predictive as well as mechanistic biomarkers. An extensive collection of patient samples (scar tissue and blood specimens), with well-annotated patient clinical and pathological data, are the essential components of efficient scar biobanking. Identifying predictive and mechanistic biomarkers allows for the implementation of scar therapeutics aligned to the molecular changes during scar development of the individual. This approach not only helps to prevent undertreating patients but also prevents their exposure to unnecessary treatment with side effects.

The new advances in genotyping, explicitly next-generation whole genome sequencing, high-resolution imaging technologies and proteomics approaches in finding biomarkers, all will help to identify the groups of patients that would scar more aggressively (Figure 1; Kwon et al., 2014; Arevalo et al., 2016; Jason et al., 2017). High-resolution imaging can make major contributions by allowing earlier diagnosis and predicting treatment response by visualizing target molecules-of-interest. In diseases like cancer, next-generation sequencing (NSG) is now being combined with routine clinical diagnostics. This not only helps in the diagnosis of patients, but also identifies therapeutic targets, unravels resistive mechanisms, and facilitates monitoring of the disease (Hsieh et al., 2017).

**Figure 1:**
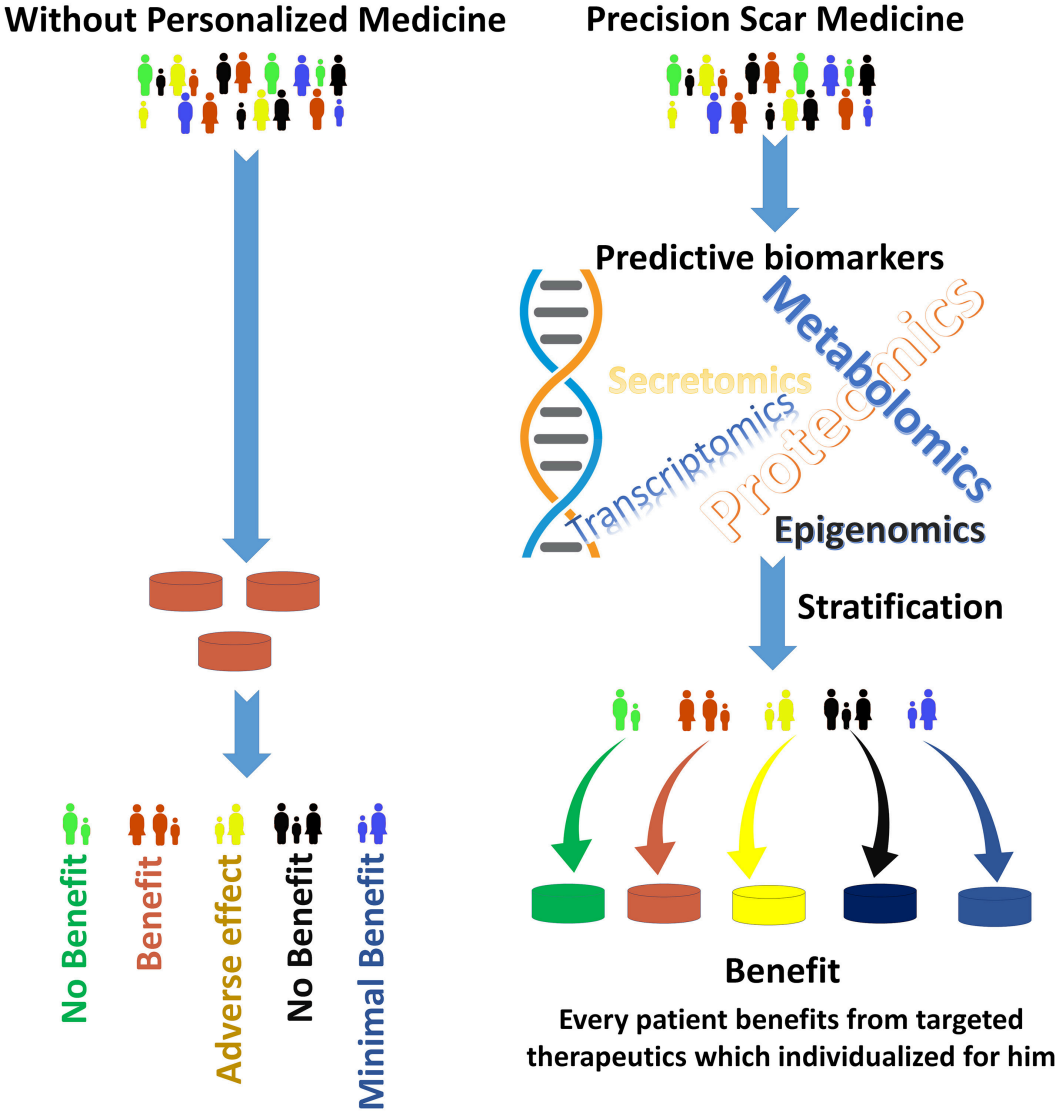
The goal of Precision Scar Medicine is to treat each patient with the best possible therapeutics. Patients with skin injuries often have different outcomes. The precision scar medicine approach provides tools to classify patients with skin injury into delicate subclasses by biomarkers that may predict responses to a particular therapeutics. Without personalized medicine **(Left)**, a “green patient” may receive brown medications which have no benefit in his scar management. Conversely, the “green patient” in the **right** will receive green therapeutics (that is, his biomarkers are associated with the best outcome with green medication) which has the maximal effect on scar prevention/management for this specific patient. The stratification methods derived from the integration of large amounts of data to find individualizing biomarkers. Additionally, this stratification may be used for the patient before elective surgeries to provide personalized preventive approaches to prevent and manage skin scars.

In therapeutics, the emerging role of stem cells during healing, their effect- or their secretome's effect, will help to develop a more personalized and stratified approach for management of skin scars. This needs an active collaboration between stem cell biologists, biomaterial scientists, clinicians, pharmaceutical, and biotech companies.

It is time to plan Precision Scar Medicine (PSM) to verify mechanistic gatekeepers and use targeted therapies to match the complexity of scar formation based on evidence-based medicine that supports clinical decision-making.

## Author contributions

The author confirms being the sole contributor of this work and approved it for publication.

### Conflict of interest statement

The author declares that the research was conducted in the absence of any commercial or financial relationships that could be construed as a potential conflict of interest.
